# 20/(fasting C-peptide × fasting plasma glucose) is a simple and effective index of insulin resistance in patients with type 2 diabetes mellitus: a preliminary report

**DOI:** 10.1186/1475-2840-12-21

**Published:** 2013-01-22

**Authors:** Tsuyoshi Ohkura, Hideki Shiochi, Youhei Fujioka, Keisuke Sumi, Naoya Yamamoto, Kazuhiko Matsuzawa, Shoichiro Izawa, Hiroshi Kinoshita, Hiroko Ohkura, Masahiko Kato, Shin-ichi Taniguchi, Kazuhiro Yamamoto

**Affiliations:** 1 Division of Cardiovascular Medicine, Endocrinology and Metabolism, Department of Molecular Medicine and Therapeutics, Tottori University Faculty of Medicine, Yonago, Tottori, Japan; 2 Department of Regional Medicine, Tottori University Faculty of Medicine, Yonago, Tottori, Japan

**Keywords:** Glucose clamp, Meal tolerance test, Japanese patients, Insulin resistance, C-peptide, Type 2 diabetes mellitus

## Abstract

**Background:**

We developed a simple and new insulin resistance index derived from a glucose clamp and a meal tolerance test (MTT) in Japanese patients with type 2 diabetes mellitus.

**Methods:**

Fifteen patients [mean age: 53 years, fasting plasma glucose (FPG) 7.7 mmol/L, HbA1c 7.1% (54 mmol/mol), body mass index 26.8 kg/m^2^] underwent a MTT and a glucose clamp. Participants were given a test meal (450 kcal). Plasma glucose and insulin were measured at 0 (fasting), 30, 60, 120, and 180 min. Serum C-peptide immunoreactivity (CPR) was measured at 0 (fasting; F-CPR) and 120 min. Homeostasis model assessment of insulin resistance (HOMA-IR) and insulin sensitivity indices (ISI) were calculated from the MTT results. The glucose infusion rate (GIR) was measured during hyperinsulinemic–euglycemic glucose clamps.

**Results:**

The mean GIR in all patients was 5.8 mg·kg^–1^·min^–1^. The index 20/(F-CPR × FPG) was correlated strongly with GIR (*r* = 0.83, *P* < 0.0005). HOMA-IR (*r* = −0.74, *P* < 0.005) and ISI (*r* = 0.66, *P* < 0.01) were also correlated with GIR. In 10 patients with mild insulin resistance (GIR 5.0–10.0 mg·kg^–1^·min^–1^), 20/(F-CPR × FPG) was very strongly correlated with GIR (*r =* 0.90, *P <* 0.0005), but not with HOMA-IR and ISI (*r = −*0.49, *P =* 0.15; *r =* 0.20, *P =* 0.56, respectively). In patients with mild insulin resistance, plasma adiponectin (*r =* 0.65, *P <* 0.05), but not BMI or waist circumstance, was correlated with GIR.

**Conclusions:**

20/(F-CPR × FPG) is a simple and effective index of insulin resistance, and performs better than HOMA-IR and ISI in Japanese patients with type 2 diabetes mellitus. Our results suggest that 20/(F-CPR × FPG) is a more effective index than HOMA-IR in Japanese patients with mild insulin resistance.

## Background

Type 2 diabetes mellitus is a heterogeneous disease characterized by insulin resistance and defective insulin secretion [1]. The most precise method to assess insulin resistance is the glucose clamp technique, although this method is very complicated and expensive [2]. The insulin sensitivity index (ISI, Matsuda–DeFronzo index) is an index of insulin resistance obtained from the glucose clamp technique and a 75-g oral glucose tolerance test (OGTT) [3]. ISI is well correlated with the glucose infusion rate (GIR) derived from the glucose clamp technique. As OGTTs should be avoided in patients with severe diabetes because of the risk of hyperglycemia, the homeostasis model assessment of insulin resistance (HOMA-IR) index is widely used in clinical practice and in clinical studies instead [4]. However, the validity of HOMA-IR may be limited in some patients, particularly those with a low BMI, decreased β cell function, and high fasting glucose levels, which are quite common in lean Korean patients with type 2 diabetes mellitus and insulin secretory defects, for example [5]. In Japan, approximately half of all patients with diabetes have a genetic predisposition to the disease, and insulin secretion is often impaired in lean patients with diabetes mellitus [6,7]. Additionally, Japanese and Asian patients often show reduced β cell function, which limits the reliability of the HOMA-IR in such populations. The quantitative insulin sensitivity check index (QUICKI) is another accurate index of insulin sensitivity and shows better correlations with the gold-standard euglycemic–hyperinsulinemic clamp method than other indices, such as the minimal model index or HOMA. However, the correlation between QUICKI and glucose clamp data is lower in nonobese subjects without diabetes than in obese subjects or patients with type 2 diabetes mellitus [8].

Insulin and C-peptide are co-secreted from the pancreas in an equimolar ratio. This phenomenon has been exploited to assess prehepatic insulin secretion in humans [9]. Unlike insulin, C-peptide is not significantly cleared by the liver and the kinetics of C-peptide are linear at physiological and supraphysiologic plasma C-peptide concentrations [10]. Therefore, it has been suggested that peripheral C-peptide levels more closely reflect pancreatic insulin secretion than do peripheral insulin levels. Moreover, although OGTTs should not be performed in patients with diabetes, meal tolerance tests (MTT) can be performed in these patients. One study reported that stimulated serum C-peptide levels during a mixed-meal tolerance test are a gold-standard measure of endogenous insulin secretion [11]. Based on these results, we hypothesized that the insulin resistance index based on C-peptide levels may be superior to an index based on insulin levels measured during a MTT. Therefore, we evaluated a new insulin resistance index based on C-peptide levels measured during a MTT and glucose clamps in Japanese patients with type 2 diabetes mellitus.

## Methods

### Subjects

Fifteen outpatients with type 2 diabetes mellitus participated in this study at Tottori University Hospital between 2009 and 2012. Type 2 diabetes mellitus was diagnosed based on the criteria of the American Diabetes Association [12]. There were eight males and seven females. The mean age of the patients was 53.2 years, mean BMI was 26.8 kg/m^2^, mean waist circumference was 92.8 cm, mean fasting plasma glucose was 7.66 mmol/L, mean HbA1c was 7.10% (54 mmol/mol), mean triglyceride was 2.60 mmol/L, and mean high-density lipoprotein cholesterol (HDL-C) was 1.40 mmol/L (Table 1). Patients with pancreatic disease, liver disease, renal failure, or those taking diabetogenic medications such as corticosteroids were excluded from this study. Three patients were on diet therapy alone; 12 were using oral hypoglycemic agents (OHAs), including α-glucosidase inhibitors (5 patients), dipeptidyl peptidase inhibitors (4) sulfonylurea (3), glinides (3), and biguanides (2). None of the patients were using thiazolidinediones or insulin.

**Table 1:** Patient characteristics

	**All patients**	**Patients with mild insulin resistance (GIR 5–10 mg kg**^**–1 **^**min**^**–1**^**)**
*n*	15	10
Sex (male/female)	8/7	6/4
Age (years)	53.2 ± 12.7	53.2 ± 15.2
BMI (kg/m^2^)	26.8 ± 2.7	25.5 ± 2.3
Waist circumstance (cm)	92.8 ± 6.7	91.7 ± 7.9
HbA1c (NGSP) (%)	7.10 ± 0.66	6.90 ± 0.64
(mmol/mol)	(55 ± 11)	(53 ± 15)
FPG (mmol/L)	7.66 ± 1.29	7.37 ± 1.06
TG (mmol/L)	2.60 ± 2.91	2.86 ± 3.59
HDL-C (mmol/L)	1.40 ± 0.35	1.47 ± 0.56
Adiponectin (μg/mL)	7.86 ± 3.00	8.20 ± 3.64
GIR (mg·kg^–1^·min^–1^)	5.80 ± 2.21	6.38 ± 1.50

Data are means ± standard deviation.

*GIR* glucose infusion rate, *BMI* body mass index, *HbA1c* hemoglobin A1c, *NGSP* National Glycohemoglobin Standardization Program, *FPG* fasting plasma glucose, *TG* triglyceride, *HDL-C* high-density lipoprotein cholesterol.

This study was approved by the Ethics Committee of the Faculty of Medicine, Tottori University. Informed consent was obtained from all of the patients using a procedure approved by the Ethics Committee.

### Meal tolerance test

After fasting for at least 12 h, the participants visited the clinic in the morning and consumed a test meal prepared by the Japan Diabetes Society (450 kcal/1882 kJ; 15% protein, 35% fat, and 50% carbohydrate; 1.6 g salt 1.6 g) [13]. Plasma glucose and insulin were measured at 0 (fasting), 30, 60, 120, and 180 min after the test meal. Serum C-peptide immunoreactivity (CPR) was measured at 0 (fasting) and 120 min. Plasma glucose was measured using the glucose oxidase method. Plasma insulin and CPR levels were measured using chemiluminescent immunoassays (CLIA) (human insulin and CPR CLIA kits; Kyowa Medix, Tokyo, Japan). Plasma insulin was defined as immunoreactive insulin (IRI). Plasma adiponectin was measured using an enzyme-linked immunosorbent assay (ELISA) (human adiponectin ELISA kit; Otsuka, Tokyo, Japan). HbA1c (JDS: Japan Diabetes Society) was measured by high-performance liquid chromatography and was converted to National Glycohemoglobin Standardization Program (NGSP) values using the following officially certified equation: NGSP (%) = 1.02 × JDS (%) + 0.25% [14]. The reverse equation is: JDS (%) = 0.980 × NGSP (%) − 0.245%. HbA1c (NGSP) values were also converted to International Federation of Clinical Chemistry (IFCC) values (mmol/mol) using the HbA1c converter developed by Diabetes UK (Macleod House, London, UK).

### Euglycemic–hyperinsulinemic clamp

Glucose clamp studies were performed 2 days after the MTT. The patients were examined in the morning after an overnight fast. An antecubital vein was cannulated to administer the infusate. A dorsal vein was cannulated and kept warm to facilitate venous sampling and provide arterialized venous blood. Using an artificial endocrine pancreas (STG 22; Nikkiso, Shizuoka, Japan), the euglycemic–hyperinsulinemic clamp was performed to determine insulin sensitivity in the peripheral tissues [2]. A primed constant infusion of insulin (100 mU/m^2^·min) and computer-controlled exogenous infusion of glucose solution were used to achieve steady-state plasma insulin levels and maintain plasma glucose (PG) levels at 5.2 mmol/L (95 mg/dL). Using an identical insulin infusion protocol, the steady-state plasma insulin level was previously reported to be 1200 pmol/L in patients with type 2 diabetes mellitus [15,16]. The steady-state GIR was calculated at 90–120 min, and the mean GIR during that time was used as a marker of peripheral insulin sensitivity.

In a previous report, a GIR > 10.0 mg·kg^–1^·min^–1^ at an insulin infusion rate of 100 mU/m^2^·min was considered normal [17]. In another study, a GIR < 5.0 mg·kg^–1^·min^–1^ was defined as insulin-resistant [18]. Therefore, in this study, we defined a GIR of 5.0–10.0 mg·kg^–1^·min^–1^ as mild insulin resistance.

### Calculation of insulin resistance indices

HOMA-IR [4] = [fasting plasma glucose (FPG; mmol/L)] × [fasting IRI (F-IRI; pmol/L)]/135. The normal range for HOMA-IR is < 2.5 [19].

ISI [3] = 10,000/√{[FPG (mmol/L) × FPI (pmol/L)] × [mean glucose × mean insulin during the MTT]}. The normal range for ISI is > 2.5 [20].

QUICKI [8] = 1/[log(HOMA-IR)] = 1/log{[FPG (mmol/L)] × [FPI (pmol/L)]/135}.

ΣIRI_(0–180)_[21] = IRI (0 min) + IRI (30 min) + IRI (60 min) + IRI (120 min) + IRI (180 min).

C-peptide index (CPI) [22] = fasting CPR (F-CPR; mmol/L)/FPG (mmol/L) × 100

Clamp-like index (CLIX) [23] = serum creatinine (mg/mL) (×0.85 if male)/{(mean AUC glucose (mg/dl) × mean AUC C-peptide (ng/mL)} × 6,600 (AUC = area under the curve). In this study, we used glucose levels at 0, 30, 60, and 120 min, and CPR levels at 0 and 120 min.

TG/HDL [24] = triglyceride (mmol/L)/high-density lipoprotein (mmol/L).

### Statistical analysis

Data are expressed as means ± standard error of the mean. Correlations between parametric clinical variables and GIR were determined by Pearson’s correlation analysis. We also calculated the intraclass correlation coefficient (ICC) to assess the agreement or consistency between pairs of indices [25]. We calculated partial correlation coefficients between GIR and 20/(F-CPR × FPG) with adjustment for the type of hypoglycemic drug/diet therapy. Values of *P* < 0.05 were considered significant. SPSS software version 15.0 (SPSS, Chicago, IL, USA) was used for all analyses.

## Results

During the steady state of glucose clamps, the mean GIR of all patients was 5.80 mg·kg^–1^·min^–1^ and the mean insulin level was 1180 ± 282 pmol/L. The mean adiponectin level was 7.86 μg/mL. In the MTT, the mean plasma glucose levels at 0 and 120 min were 7.66 mmol/L and 10.10 mmol/L, respectively (Table 2). The mean values for HOMA-IR, ISI, QUICKI, CPI, and CLIX were 2.90, 5.30, 0.30, 8.30, and 8.50, respectively. Although we tested various indices based on glucose, insulin, and C-peptide levels, the correlation was strongest between GIR and 20/(F-CPR × FPG). The mean value for 20/(F-CPR × FPG) in the study patients was 5.00.

**Table 2:** Results of the meal tolerance test

**Time (min)**	**0**	**30**	**60**	**120**	**180**
Glucose (mmol/L)	7.66 ± 1.29	8.96 ± 2.34	10.24 ± 3.12	10.10 ± 2.52	8.50 ± 2.47
Insulin (pmol/L)	60.5 ± 39.7	180.0 ± 141.0	220.9 ± 135.5	263.7 ± 147.8	216.3 ± 170.0
CPR (nmol/L)	0.60 ± 0.25			1.60 ± 0.48	
**Insulin resistance indices calculated from the MTT**					
Indices	HOMA-IR	ISI	QUICKI	CPI	20(F-CPR × FPG)
Value	2.90 ± 1.84	5.30 ± 2.20	0.30 ± 0.02	8.30 ± 4.21	5.00 ± 2.00

Data are means ± standard deviation.

*CPR* C-peptide immunoreactivity, *MTT* meal tolerance test, *HOMA-IR* homeostasis model assessment of insulin resistance, *ISI* insulin sensitivity index, *QUICKI* quantitative insulin sensitivity check index, *CPI* C-peptide index, *F-CPR* fasting C-peptide immunoreactivity, *FPG* fasting plasma glucose.

**Figure 1:**
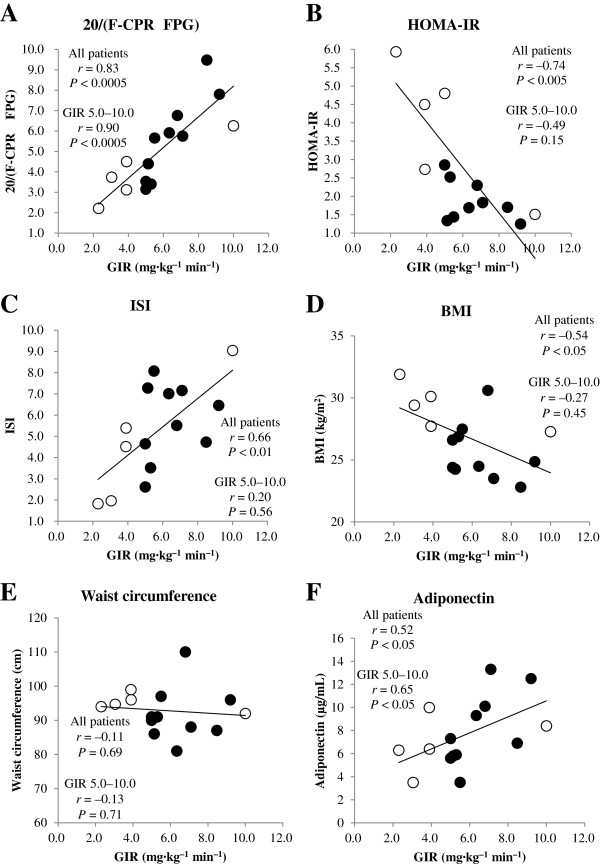
**Correlation between insulin resistance indices and GIR.** Correlations between GIR and 20/(F-CPR × FPG) (**A**), HOMA-IR (**B**), ISI (**C**), BMI (**D**), waist circumstance (**E**), and adiponectin (**F**) were calculated by simple regression analysis. Black circles = patients with GIR 5–10 (mg·kg^–1^·min^–1^); white circles = patients with GIR < 5 or > 10 mg·kg^–1^·min^–1^.

Both F-CPR × FPG (*r* = −0.81, *P* < 0.0005) (Table 3) and 1/(F-CPR × FPG) were correlated with GIR (*r =* 0.83, *P <* 0.0005) (Table 3). Based on the regression equation GIR = 18.7 × 1/(F-CPR × FPG) + 1.16, we proposed the novel index 20/(F-CPR × FPG) to estimate GIR. The index 20/(F-CPR × FPG) was also strongly correlated with GIR (*r =* 0.83, *P <* 0.0005) and could estimate GIR (Table 3, Figure 1A). HOMA-IR (*r* = −0.74, *P* < 0.005) (Figure 1B) and ISI (*r* = 0.66, *P* < 0.01) were both correlated with GIR (Figure 1C).

**Table 3:** Correlation coefficients between indices of insulin resistance and glucose infusion rate during the meal tolerance test

**Index**	**All patients**		**GIR 5–10 mg·kg**^**–1**^**·min**^**–1**^	
	**(*n *= 15)**		**(n = 10)**	
	*r*	*P*	*r*	*P*
HOMA-IR	−0.74	< 0.005	−0.49	0.15
ISI	0.66	< 0.01	0.20	0.56
QUICKI	0.76	< 0.001	0.50	0.14
F-IRI	−0.66	< 0.01	−0.42	0.22
ΣIRI_(0–180 min)_	−0.59	< 0.05	−0.26	0.54
F-CPR	−0.71	< 0.01	−0.75	< 0.05
F-CPR × FPG	−0.81	< 0.0005	−0.83	< 0.005
1/(F-CPR × FPG)	0.83	< 0.0005	0.90	< 0.0005
20/(F-CPR × FPG)	0.83	< 0.0005	0.90	<0.0005
CPI	−0.52	< 0.05	−0.62	0.06
CLIX	0.40	0.12	0.23	0.52
P-CPR × PPG	−0.58	< 0.05	−0.48	0.16
(F-CPR + P-CPR) × (FPG + PPG)	−0.68	< 0.005	−0.63	< 0.05
BMI	−0.54	< 0.05	−0.27	0.45
Waist circumference	−0.11	0.69	−0.13	0.71
TG/HDL	−0.11	0.67	−0.32	0.35
Plasma adiponectin	0.52	< 0.05	0.65	< 0.05

Correlation coefficients were determined using Pearson’s product moment correlation coefficient test.

*GIR* glucose infusion rate, *HOMA-IR* homeostasis model assessment of insulin resistance, *ISI* insulin sensitivity index, *QUICKI* quantitative insulin sensitivity check index, *F-IRI* fasting immunoreactive insulin, *F-CPR* fasting C-peptide immunoreactivity, *FPG* fasting plasma glucose, *CPI* C-peptide index, *P-CPR* 2-h postprandial C-peptide immunoreactivity, *PPG* 2-h postprandial plasma glucose, *BMI* body mass index, *TG* triglyceride, *HDL-C* high-density lipoprotein cholesterol.

In 10 patients with mild insulin resistance, defined as GIR 5–10 mg·kg^–1^·min^–1^, 20/(F-CPR × FPG) was strongly correlated with GIR (*r =* 0.90, *P <* 0.0005), whereas HOMA-IR (*r* = −0.49, *P* = 0.15) and ISI (*r* = 0.20, *P* = 0.56) were not. QUICKI was also strongly correlated with GIR in all patients (*r* = 0.76, *P* < 0.001), but not in patients with mild insulin resistance (*r* = 0.50, *P* = 0.14). Similarly, CPI was significantly correlated with GIR in all patients (*r* = −0.52, *P* < 0.05), but not in patients with the mild insulin resistance (*r* = −0.62, *P* = 0.06). CLIX was not correlated with GIR in all patients (*r* = 0.40, *P* = 0.12), nor in patients with mild insulin resistance (*r* = 0.23, *P* = 0.52). F-CPR (*r* = −0.71, *P* < 0.01) and F-IRI (*r* = −0.66, *P* < 0.01) were correlated with GIR in all patients. Among patients with mild insulin resistance, F-CPR was significantly correlated with GIR (*r* = −0.75, *P* < 0.05), whereas F-IRI was not (*r* = −0.42, *P* = 0.22). ΣIRI_(0–180)_ was significantly correlated with GIR in all patients (*r* = −0.59, *P* < 0.05), but not in patients with mild insulin resistance (*r* = −0.26, *P* = 0.54).

In terms of other clinical factors, BMI was significantly correlated with GIR in all patients (*r* = −0.54, *P* < 0.05), but not in patients with mild insulin resistance (*r* = −0.27, *P* = 0.45; Figure 1D). By contrast, waist circumstance was not correlated with GIR in all patients (*r* = −0.11, *P* = 0.69), nor in patients with mild insulin resistance (*r* = −0.13, *P* = 0.71) (Figure 1E). TG/HDL was not correlated with GIR in all patients (*r* = −0.11, *P* = 0.67), nor in patients with mild insulin resistance (*r* = −0.32, *P* = 0.35). Plasma adiponectin was correlated with GIR in all patients (*r* = 0.52, *P* < 0.05), and the correlation was greater in patients with mild insulin resistance (*r* = 0.65, *P* < 0.05; Figure 1F).

We also analyzed calculated the ICC between specific indices and GIR. The ICC between 20/(F-CPR × FPG) was 0.828 (*P <* 0.0001) in all patients and 0.859 (*P <* 0.0001) in patients with mild insulin resistance. ICC between GIR and other indices in all patients and patients with mild insulin resistance were −0.724 (*P <* 0.001) and −0.456 (*P =* 0.079), respectively, for HOMA-IR, and 0.665 (*P <* 0.005) and 0.198 (*P =* 0.279), respectively, for ISI.

Next, we determined partial correlation coefficients between 20/(F-CPR × FPG) and GIR after adjusting for the use of hypoglycemic drugs/diet therapy. The correlation between 20/(F-CPR × FPG) and GIR remained significant in all of the analyses (Table 4).

**Table 4:** Partial correlation coefficients between 20/(F-CPR × FPG) and GIR adjusted for the use of hypoglycemic drugs/diet therapy

**Hypoglycemic drug/diet therapy**	**All patients**		**GIR 5–10 mg·kg**^**–1**^**·min**^**–1**^	
	**(*n *= 15)**		**(n = 10)**	
	**Partial *r***	***P***	**Partial *r***	***P***
All drugs	0.854	< 0.0001	0.917	< 0.0001
Biganide	0.830	< 0.0001	0.902	< 0.0001
Sulfonylurea	0.839	< 0.0001	0.949	< 0.0001
DPP4 inhibitor	0.834	< 0.0001	0.896	< 0.0001
Glinide	0.833	< 0.0001	0.886	< 0.0001
α-GI	0.793	0.001	0.889	< 0.0001
Diet therapy alone	0.854	< 0.0001	0.917	< 0.0001

Correlations between 20/(F-CPR × FPG) and GIR were adjusted for all drugs or for individual drugs.

*DPP4* dipeptidyl peptidase 4, *GI* glucosidase inhibitor.

We also tested the index 2-h postprandial CPR (P-CPR) × 2-h postprandial plasma glucose (PPG) (P-CPR × PPG). P-CPR × PPG was significantly correlated with GIR in all patient (*r* = −0.58, *P* < 0.05) and showed a tendency to be correlated with GIR in patients with mild insulin resistance (*r* = −0.48, *P* = 0.16). Another index, (F-CPR + P-CPR) × (FPG + PPG) was significantly correlated with GIR in all patients (*r* = −0.68 *P* < 0.005) and in patients with mild insulin resistance (*r* = −0.63, *P* < 0.05).

There were four patients with insulin resistance, which was defined as GIR <5 mg·kg^–1^·min^–1^. All four patients had HOMA-IR > 2.5, the cutoff for insulin resistance, but only two patients had ISI < 2.5, another definition of insulin resistance. Of 10 patients with mild insulin resistance (GIR 5–10 mg·kg^–1^·min^–1^), only three had HOMA-IR > 2.5 and none had ISI <2.5.

## Discussion

This study revealed that the index 20/(F-CPR × FPG) was more strongly correlated with GIR than were HOMA-IR, QUICKI, ISI, and CPI. Of note, 20/(F-CPR × FPG) was also correlated with GIR in patients with mild insulin resistance, unlike HOMA-IR, QUICKI, and ISI. HOMA-IR is simple, and the most commonly used index of insulin resistance. However, HOMA-IR is of limited use in subjects with a lower BMI, decreased β cell function, and high FPG levels [5]. As Japanese and Asian populations often show reduced β cell function, HOMA-IR is unsuitable in these populations [6,7]. In our study, HOMA-IR was not correlated with GIR in patients with mild insulin resistance. QUICKI was also reported to show weaker correlations with GIR recorded during glucose clamp in nonobese subjects than in obese subjects [8], with similar results in our study. Using the hyperglycemic clamp technique to study glucose tolerance in normotensive Americans, Chiu et al. reported that Caucasians were more insulin sensitive than Asian-Americans, and that their β cells compensated for the prevailing insulin sensitivity [26].

Asian-Americans appear to have an ethnic propensity to insulin resistance that is not explained by obesity. For example, single nucleotide polymorphisms in the adiponectin gene in Japanese subjects are associated with insulin resistance and type 2 diabetes mellitus, and may be mediated by changes in the expression and plasma concentrations of adiponectin [27]. Matsuhisa et al. reported that a modified version of HOMA-IR, termed HOMA-AD, was correlated with the results of glucose clamp studies. HOMA-AD was calculated as (serum insulin × plasma glucose)/serum adiponectin [28]. BMI and waist circumference were not correlated with GIR, but plasma adiponectin was correlated with GIR in patients with mild insulin resistance in our study. Therefore, we think that adiponectin is an important mediator of insulin resistance in Asian individuals. In earlier studies, individuals with HOMA-IR > 2.5 [19] or ISI < 2.5 [20] were classified as having insulin resistance. Of 10 patients with mild insulin resistance (GIR 5–10 mg·kg^–1^·min^–1^), only three had HOMA-IR > 2.5 and none had ISI < 2.5. Therefore, these indices and the currently accepted ranges are not appropriate for identifying patients with mild insulin resistance. Consequently, we suggest that HOMA-IR and ISI are not suitable for Japanese and other Asian populations.

Insulin and C-peptide are co-secreted in an equimolar ratio by the pancreas. Unlike insulin, C-peptide is not cleared by the liver, resulting in a longer half-life [9,10]. Accordingly, we think that an index incorporating C-peptide levels will perform better than an index based on insulin levels. Indeed, among patients with mild insulin resistance, the index 20/(F-CPR × FPG) was more strongly correlated with GIR than were HOMA-IR and ISI. \These results suggest that plasma C-peptide levels better reflect insulin bioactivity in skeletal muscle, as the glucose clamp technique mainly reflects insulin resistance in skeletal muscle. 20/(F-CPR × FPG) also showed stronger correlations with GIR than did CPI, an index of insulin secretory ability [22]. Therefore, we consider that 20/(F-CPR × FPG) is superior to CPI for evaluating insulin resistance.

As the index (F-CPR + P-CPR) × (FPG + PPG) was also more effective than ISI in patients with mild insulin resistance, we think it provides a simple and effective index of insulin resistance during a MTT. However, as 20/(F-CPR × FPG) requires a single blood sample and does not require a MTT, we recommend this index for screening of insulin resistance.

Our study had several limitations, including the small number of patients and the variable nature of the medications of diabetes used by the study participants. As only 15 patients participated in this study, our results require confirmation in a larger study. We also think that the coefficient “20” included in our index requires further examination to confirm its validity. Indeed, we are currently conducting a larger study, the results of which we plan to publish in the future. It is possible that the different medications used by the subjects modified the insulin and C-peptide responses in the MTT. Moreover, as ISI and CLIX were originally obtained from an OGTT, the differences between the test meal and glucose load may also affect insulin levels. By contrast, CPR levels accurately reflected insulin resistance determined during a glucose clamp, despite the variability in hypoglycemic drugs used by our subjects. Such variations are often experienced in daily clinical work.

Based on an earlier report, we excluded patients with serum creatinine > 1.3 mg/dl [29]. If we use our index in patients with renal insufficiency, a corrective may be needed in the equation, as with CLIX. As C-peptide secretion is generally lower in patients after pancreas transplantation, our index may not be suitable for patients who have undergone pancreas transplantation.

Thigh and calf circumferences are correlated with insulin resistance in patients with type 2 diabetes mellitus [30]. Unfortunately, we did not measure thigh or calf circumferences, which should be done in future studies.

Despite these limitations, we think that our study and the new index may aid routine clinical treatment of Japanese and other Asian patients with type 2 diabetes mellitus, particularly because 20/(F-CPR × FPG) is very easy to calculate using a single blood sample.

## Conclusion

20/ (F-CPR × FPG) is a simple and effective index of insulin resistance compared with HOMA-IR and ISI in Japanese patients with type 2 diabetes mellitus. In particular, 20/(F-CPR × FPG) showed a stronger correlation with GIR than did HOMA-IR in patients with mild insulin resistance. Furthermore, it is possible to predict GIR by calculating 20/(F-CPR × FPG). As Japanese and other Asian patients do not generally show hyperinsulinemia, we think that our index is particularly effective in these populations.

## Abbreviations

BMI: Body mass index; CLIX: Clamp-like index; CPI: C-peptide index; CPR: C-peptide immunoreactivity; F-CPR: Fasting C-peptide immunoreactivity; F-IRI: Fasting immunoreactive insulin; FPG: Fasting plasma glucose; GIR: Glucose infusion rate; HDL-C: High-density lipoprotein cholesterol; HOMA-IR: Homeostasis model assessment for insulin resistance; IRI: Immunoreactive insulin; ISI: Insulin sensitivity index; MTT: Meal tolerance test; P-CPR: Postprandial C-peptide immunoreactivity; PPG: Postprandial plasma glucose; QUICKI: Quantitative insulin sensitivity check index; TG: Triglyceride.

## Competing interests

The authors declare that they have no competing interests.

## Authors’ contributions

TO participated in the design of the study and performed the statistical analysis. HS, YF, KS, NY, KM, SI, HK, and HO collected the data. MK, ST, and KY conceived the study, participated in its design and coordination, and helped to draft the manuscript. All authors read and approved the final manuscript.

## References

[B1] DeFronzo RA Lilly lecture 1987. The triumvirate: beta-cell, muscle, liver. A collusion responsible for NIDDM Diabetes 1988 37 667 687 3289989 10.2337/diab.37.6.667

[B2] DeFronzo RA Tobin JD Andres R Glucose clamp technique: a method for quantifying insulin secretion and resistance Am J Physiol 1979 237 214 223 10.1152/ajpendo.1979.237.3.E214 382871

[B3] Matsuda M DeFronzo RA Insulin sensitivity indices obtained from oral glucose tolerance testing: comparison with the euglycemic insulin clamp Diabetes Care 1999 22 1462 1470 10.2337/diacare.22.9.1462 10480510

[B4] Matthews DR Hosker JP Rudenski AS Naylor BA Treacher DF Turner RC Homeostasis model assessment: insulin resistance and beta-cell function from fasting plasma glucose and insulin concentrations in man Diabetologia 1985 28 412 419 10.1007/BF00280883 3899825

[B5] Kang ES Yun YS Park SW Kim HJ Ahn CW Song YD Cha BS Lim SK Kim KR Lee HC Limitation of the validity of the homeostasis model assessment as an index of insulin resistance in Korea Metabolism 2005 54 206 211 10.1016/j.metabol.2004.08.014 15690315

[B6] Kadowaki T Miyake Y Kajinuma H Risk factors for worsening to diabetes in subjects with impaired glucose tolerance Diabetologia 1984 26 44 49 6368299 10.1007/BF00252262

[B7] Kadowaki T Yoshinaga H Risk factors for the development of non-insulin-dependent diabetes mellitus (NIDDM) in Japan Diabetes Res Clin Pract 1994 24 123 127 10.1016/0168-8227(94)90238-0 7859593

[B8] Katz A Nambi SS Mather K Baron AD Follmann DA Sullivan G Quon MJ Quantitative insulin sensitivity check index: a simple, accurate method for assessing insulin sensitivity in humans J Clin Endocrinol Metab 2000 85 2402 2410 10.1210/jc.85.7.2402 10902785

[B9] Eaton RP Allen RC Schade DS Erickson KM Standefer J Prehepatic insulin production in man: kinetic analysis using peripheral connecting peptide behaviour J Clin Endocrinol Metab 1980 51 520 528 10.1210/jcem-51-3-520 6997329

[B10] Polonsky KS Licinio-Paixao J Given BD Pugh W Rue P Galloway J Karrison T Frank B Use of biosynthetic human C-peptide in the measurement of insulin secretion rates in normal volunteers and type I diabetic patients J Clin Invest 1986 77 98 105 10.1172/JCI112308 3511094 PMC423314

[B11] Greenbaum CJ Mandrup-Poulsen T McGee PF Battelino T Haastert B Ludvigsson J Pozzilli P Lachin JM Kolb H Type 1 Diabetes Trial Net Research Group; European C-Peptide Trial Study Group Mixed-meal tolerance test versus glucagon stimulation test for the assessment of beta-cell function in therapeutic trials in type 1 diabetes Diabetes Care 2008 31 1966 1971 10.2337/dc07-2451 18628574 PMC2551636

[B12] American Diabetes Association Diagnosis and classification of diabetes mellitus Diabetes Care 2007 30 42 47

[B13] Yoshino G Tominaga M Hirano T Shiba T Kashiwagi A Tanaka A Tada N Onuma T Egusa G Kuwashima M Sanke T Oikawa S Honda K Tachikawa T The test meal A:A pilot model for the international standard of test meal for an assessment of both postprandial hyperglycemia and hyperlipidemia J Jpn Diabetes Soc 2006 49 361 371

[B14] Kashiwagi A Kasuga M Araki E Oka Y Hanafusa T Ito H Tominaga M Oikawa S Noda M Kawamura T Sanke T Namba M Hashiramoto M Sasahara T Nishio Y Kuwa K Ueki K Takei I Umemoto M Murakami M Yamakado M Yatomi Y Ohashi H Committee on the Standardization of Diabetes Mellitus-Related Laboratory Testing of Japan Diabetes Society, “International clinical harmonization of glycated hemoglobin in Japan: From Japan Diabetes Society to National Glycohemoglobin Standardization Program values J Diabetes Invest 2012 3 39 40 10.1111/j.2040-1124.2012.00207.x PMC4014931 24843544

[B15] Kawamori R Matsuhisa M Kinoshita J Mochizuki K Niwa M Arisaka T Ikeda M Kubota M Wada M Kanda T Ikebuchi M Tohdo R Yamasaki Y Pioglitazone enhances splanchnic glucose uptake as well as peripheral glucose uptake in non-insulin-dependent diabetes mellitus. AD-4833 Clamp-OGL Study Group Diabetes Res Clin Pract 1998 41 35 43 10.1016/S0168-8227(98)00056-4 9768370

[B16] Tamura Y Tanaka Y Sato F Choi JB Watada H Niwa M Kinoshita J Ooka A Kumashiro N Igarashi Y Kyogoku S Maehara T Kawasumi M Hirose T Kawamori R Effects of diet and exercise on muscle and liver intracellular lipid contents and insulin sensitivity in type 2 diabetic patients J Clin Endocrinol Metab 2005 90 3191 3196 10.1210/jc.2004-1959 15769987

[B17] Sakurai Y Tamura Y Takeno K Kumashiro N Sato F Kakehi S Ikeda S Ogura Y Saga N Naito H Katamoto S Fujitani Y Hirose T Kawamori R Watada H Determinants of intramyocellular lipid accumulation after dietary fat loading in non-obese men J Diabetes Invest 2011 4 310 317 10.1111/j.2040-1124.2010.00091.x PMC4014973 24843504

[B18] Sato F Tamura Y Watada H Kawamori R Kumashiro N Igarashi Y Uchino H Maehara T Kyogoku S Sunayama S Sato H Hirose T Tanaka Y Kawamori R Effects of diet-induced moderate weight reduction on intrahepatic and intramyocellular triglycerides and glucose metabolism in obese subjects J Clin Endocrinol Metab 2007 92 3326 3329 10.1210/jc.2006-2384 17519317

[B19] Bonora E Kiechl S Willeit J Muggeo M Prevalence of insulin resistance in metabolic disorders: the Bruneck Study Diabetes 1998 47 1643 1649 10.2337/diabetes.47.10.1643 9753305

[B20] Kernan WN Inzucchi SE Viscoli CM Brass LM Bravata DM Shulman GI McVeety JC Horwitz RI Pioglitazone improves insulin sensitivity among nondiabetic patients with a recent transient ischemic attack or ischemic stroke Stroke 2003 34 1431 1436 10.1161/01.STR.0000071108.00234.0E 12730556

[B21] Yamada N Yoshinaga H Sakurai N Shimano H Gotoda T Ohashi Y Yazaki Y Kosaka K Increased risk factors for coronary artery disease in Japanese subjects with hyperinsulinemia or glucose intolerance Diabetes Care 1994 17 107 114 10.2337/diacare.17.2.107 8137680

[B22] Iwata M Maeda S Kamura Y Takano A Kato H Murakami S Higuchi K Takahashi A Fujita H Hara K Kadowaki T Tobe K Genetic risk score constructed using 14 susceptibility alleles for type 2 diabetes is associated with the early onset of diabetes and may predict the future requirement of insulin injections among Japanese individuals Diabetes Care 2012 35 1763 1770 10.2337/dc11-2006 22688542 PMC3402252

[B23] Anderwald C Anderwald-Stadler M Promintzer M Prager G Mandl M Nowotny P Bischof MG Wolzt M Ludvik B Kästenbauer T Pacini G Luger A Krebs M The Clamp-Like Index: a novel and highly sensitive insulin sensitivity index to calculate hyperinsulinemic clamp glucose infusion rates from oral glucose tolerance tests in nondiabetic subjects Diabetes Care 2007 30 2374 2380 10.2337/dc07-0422 17595351

[B24] Chiang JK Lai NS Chang JK Koo M Predicting insulin resistance using the triglyceride-to-high-density lipoprotein cholesterol ratio in Taiwanese adults Cardiovasc Diabetol 2011 10 93 10.1186/1475-2840-10-93 22004541 PMC3224454

[B25] Lee J Koh D Ong CN Statistical evaluation of agreement between two methods for measuring a quantitative variable Comput Biol Med 1989 19 61 70 10.1016/0010-4825(89)90036-X 2917462

[B26] Chiu KC Cohan P Lee NP Chuang LM Insulin sensitivity differs among ethnic groups with a compensatory response in beta-cell function Diabetes Care 2000 23 1353 1358 10.2337/diacare.23.9.1353 10977032

[B27] Hara K Boutin P Mori Y Tobe K Dina C Yasuda K Yamauchi T Otabe S Okada T Eto K Kadowaki H Hagura R Akanuma Y Yazaki Y Nagai R Taniyama M Matsubara K Yoda M Nakano Y Tomita M Kimura S Ito C Froguel P Kadowaki T Genetic variation in the gene encoding adiponectin is associated with an increased risk of type 2 diabetes in the Japanese population Diabetes 2002 51 536 540 11812766 10.2337/diabetes.51.2.536

[B28] Matsuhisa M Yamasaki Y Emoto M Shimabukuro M Ueda S Funahashi T Matsuzawa Y A novel index of insulin resistance determined from the homeostasis model assessment index and adiponectin levels in Japanese subjects Diabetes Res Clin Pract 2007 77 151 154 10.1016/j.diabres.2006.10.005 17081646

[B29] Funakoshi S Fujimoto S Hamasaki A Fujiwara H Fujita Y Ikeda K Hamamoto Y Hosokawa M Seino Y Inagaki N Analysis of factors influencing pancreatic beta-cell function in Japanese patients with type 2 diabetes: association with body mass index and duration of diabetic exposure Diabetes Res Clin Pract 2008 82 353 358 10.1016/j.diabres.2008.09.010 18950889

[B30] Park JS Cho MH Ahn CW Kim KR Huh KB The association of insulin resistance and carotid atherosclerosis with thigh and calf circumference in patients with type 2 diabetes Cardiovasc Diabetol 2012 11 62 10.1186/1475-2840-11-62 22682537 PMC3444381

